# Association between physical activity level and frailty status among community-dwelling older adults with multimorbidity: a cross-sectional study

**DOI:** 10.3389/fpubh.2026.1826097

**Published:** 2026-07-01

**Authors:** Rongjing Wang, Ning Yang, Jie Wang, Galiya Madiyeva, Wei Lyu, Wei Sun

**Affiliations:** 1 Shandong Management University, Jinan, China; 2 Shandong Youth University of Political Science, Jinan, China; 3 Qufu Normal University, Qufu, China; 4 Faculty of Medicine and Health, Al-Farabi Kazakh National University, Almaty, Kazakhstan; 5 Beijing City University, Beijing, China

**Keywords:** cross-sectional study, frailty, multimorbidity, older adults, physical activity

## Abstract

**Objective:**

To examine the cross-sectional association between physical activity characteristics and frailty status among community-dwelling older adults with multimorbidity, and to provide descriptive evidence for community-level health promotion in this high-risk population.

**Methods:**

A cross-sectional survey was conducted among 500 community-dwelling older adults with multimorbidity. Frailty was assessed using the FRAIL scale and classified into three groups: non-frail, pre-frail, and frail. Physical activity was evaluated using the Rapid Assessment of Physical Activity (RAPA) questionnaire and analyzed across three dimensions: aerobic activity level, strength training, and flexibility exercise. Between-group differences were compared using the chi-square test and the Kruskal–Wallis test. Ordinal logistic regression was used to examine the association between physical activity characteristics and frailty status. Spearman correlation analysis and linear regression were performed as sensitivity analyses.

**Results:**

Significant differences were observed in the distribution of frailty status across physical activity levels, with a clear linear trend (all *p* < 0.001). In the multivariable analysis, compared with individuals with low activity levels, those who were regularly active but did not meet the recommended level were significantly less likely to be classified into a higher frailty category (OR = 0.23, 95% CI: 0.14–0.39), and this likelihood was further reduced among those who met the recommended activity level (OR = 0.11, 95% CI: 0.06–0.21); the trend test was statistically significant (P for trend < 0.001). Using binary participation indicators, neither strength training nor flexibility exercise showed an independent statistical association with frailty status after adjustment. Sensitivity analyses indicated a moderate negative correlation between physical activity score and total frailty score (rho = −0.454, *p* < 0.001), which remained significant in linear regression models. This association persisted after further adjustment for disease burden, disease-system count, and disease-system indicators. Exploratory analyses considering disease burden and disease-system categories showed that the inverse association between physical activity level and frailty status remained robust.

**Conclusion:**

Among community-dwelling older adults with multimorbidity, higher physical activity levels were cross-sectionally associated with lower frailty status. Overall physical activity level may better reflect current frailty status than single exercise components. These findings support the value of promoting overall physical activity in community health management, but longitudinal studies are needed to determine whether increasing physical activity can prevent frailty onset or slow frailty progression.

## 1 Introduction

With the continued deepening of population aging, frailty has become a major public health issue in the field of healthy aging. A systematic review including more than 1.75 million participants from 62 countries reported that, when assessed using physical frailty criteria, the prevalence of frailty among older adults was approximately 12%, while the prevalence of prefrailty was about 46%, with a heavier burden observed in women and the oldest-old populations (1). In community populations, frailty is not a static condition. A global meta-analysis showed that the pooled incidence of frailty among originally non-frail older adults was 43.4 per 1,000 person-years, whereas the incidence among prefrail individuals was as high as 150.6 per 1,000 person-years (2). More importantly, frailty is closely associated with adverse outcomes such as falls, fractures, disability, dementia, hospitalization, and death (3). In the context of multimorbidity, frailty and the accumulation of chronic diseases are closely intertwined. A systematic review indicated that most frail older adults also live with multimorbidity, and a meta-analysis specifically targeting community-dwelling older adults with multimorbidity further showed that the pooled prevalence of frailty and prefrailty in this population reached 18.1% and 48.9%, respectively (4, 5).

Physical activity is one of the few health behaviors that is simultaneously modifiable, scalable, and capable of generating population-wide benefits. The 2020 WHO guidelines recommend that older adults perform 150–300 min of moderate-intensity aerobic activity or 75–150 min of vigorous-intensity aerobic activity per week, combined with regular muscle-strengthening activities, while emphasizing that “some physical activity is better than none” (6). The Physical Activity Guidelines for Americans further state that, in addition to aerobic and muscle-strengthening activities, older adults should also incorporate balance training, and that even modest increases in activity can yield substantial health benefits for the least active individuals (7). A systematic review and meta-analysis of cohort studies demonstrated that individuals with the highest levels of physical activity had a 41% lower risk of developing frailty compared with those with the lowest levels, suggesting that physical activity may be an important protective factor in delaying frailty progression (8). However, in terms of real-world implementation, only 13.9% of U.S. adults aged 65 years and older met the recommended standards for both aerobic and muscle-strengthening activities in 2022, indicating that insufficient physical activity remains common among older adults, particularly those with chronic conditions (9).

Although existing evidence has suggested a relationship between exercise and frailty, sufficiently clear evidence is still lacking regarding which specific physical activity characteristics are most critical among older adults with multimorbidity. A systematic review focusing on this population found that as many as 22 multicomponent exercise programs had been investigated, with substantial variation in frequency, intensity, and duration, while few were specifically designed around the functional capacity and participation barriers of this group (10). Another recent meta-analysis confirmed that multicomponent exercise can improve frailty status as well as functional indicators such as muscle strength, gait speed, and balance; however, such studies have largely focused on the overall effects of interventions, making it difficult to disentangle the respective contributions of aerobic activity level and individual components such as strength training and flexibility exercise (11). A systematic review involving both healthy older adults and frail older adults also noted that the benefits of different exercise modalities are not entirely consistent across outcomes, and that substantial heterogeneity remains in the current literature, highlighting the need for analyses in more clearly defined populations and across more refined dimensions of physical activity (12).

Against this background, the present study targeted community-dwelling older adults with multimorbidity and adopted a cross-sectional design to examine the association between current physical activity characteristics and current frailty status. In this study, multimorbidity was used primarily as a population-defining high-risk context rather than as a longitudinal exposure. We acknowledge that multimorbidity is dynamic and that changes in the type, number, severity, and combinations of chronic diseases over time cannot be captured in a cross-sectional survey. Previous longitudinal evidence has shown that multimorbidity patterns are differentially associated with incident physical frailty, particularly cardiovascular and neuropsychiatric patterns (13, 34). Therefore, the present study does not aim to infer causal effects on frailty onset or progression. Instead, it aims to describe whether physical activity level, strength training, and flexibility exercise are associated with frailty status within a multimorbid community population, and to further explore multimorbidity heterogeneity using disease burden and disease-system categories.

## 2 Methods

### 2.1 Study design and reporting guideline

This study employed a community-based cross-sectional design targeting community-dwelling older adults with multimorbidity. Questionnaire surveys were used to collect data on physical activity characteristics, frailty status, and related sociodemographic information, and to examine the associations between physical activity characteristics and frailty status. The study was reported in accordance with the STROBE (Strengthening the Reporting of Observational Studies in Epidemiology) statement for cross-sectional studies (14, 15), with systematic reporting of the study design, participants, variable definitions, data processing, statistical analysis, and presentation of results, in order to enhance the transparency of the research process, the standardization of result reporting, and the interpretability of the findings. No prospective follow-up assessment was conducted; therefore, the analyses focused on cross-sectional associations between current physical activity characteristics and current frailty status.

### 2.2 Study setting and participants

The study population consisted of community-dwelling older adults. The inclusion criteria were as follows: age 65 years or older; long-term residence in the community; basic communication ability and capacity to complete the questionnaire; physician-confirmed diagnosis of at least two chronic diseases (16); and provision of informed consent with voluntary participation. The exclusion criteria were as follows: missing data on key variables; obvious logical inconsistencies in the questionnaire that could not be verified; and inability to complete the main scales. After screening, a total of 500 community-dwelling older adults with multimorbidity were included in the final analysis. The study was conducted in community settings in China. Both urban and rural communities were included; however, urban/rural residence was not recorded at the individual level and therefore could not be included in subgroup or regression analyses.

### 2.3 Sample size consideration

The adequacy of the final sample size was assessed using G*Power 3.1. Based on a chi-square test for the association between three physical activity categories and three frailty status categories, with a small-to-moderate effect size of w = 0.16, a two-sided *α* of 0.05, 80% statistical power, and 4 degrees of freedom, the minimum required sample size was estimated to be 467 participants. Considering potential invalid questionnaires, missing data, and logical inconsistencies, the target sample size was increased. After data screening, 500 eligible participants were included in the final analysis, which exceeded the minimum estimated sample size.

### 2.4 Variable definitions and measurements

#### 2.4.1 Outcome variable: frailty

Frailty was assessed using the FRAIL scale. This instrument consists of five items reflecting fatigue, difficulty climbing stairs, difficulty walking, disease burden, and unintentional weight loss. Each item was scored as 1 for “yes” and 0 for “no,” yielding a total score ranging from 0 to 5, with higher scores indicating greater frailty severity. According to commonly used cut-off criteria, a total score of 0 was defined as non-frail, 1–2 as prefrail, and 3–5 as frail (17, 18). In this study, the five items were summed to generate a total frailty score (frail total), and a frailty grouping variable (frail group) was further constructed, in which 0 indicated non-frail, 1 indicated prefrail, and 2 indicated frail.

### 2.5 Main exposure variable: physical activity characteristics

Physical activity was assessed using the structure of the RAPA questionnaire. The instrument comprises nine items, of which the first seven assess aerobic activity level, and the final two reflect participation in strength training and flexibility exercise, respectively (19). For the first seven items, the physical activity level of each participant was determined according to the highest positive response, with a higher level indicating a higher overall activity level. Based on these seven items, a physical activity level variable (pa score7) was generated, ranging from 1 to 7. The physical activity level variable was collapsed into three categories: scores of 1–2 were defined as low activity, scores of 3–5 as regularly active but below the recommended level, and scores of 6–7 as meeting the recommended level. In addition, based on the last two questionnaire items, variables for strength training and flexibility exercise were created, with participation coded as 1 and non-participation coded as 0. Physical activity characteristics were therefore characterized at two levels: overall activity level and specific exercise types (20). Because the RAPA items for strength training and flexibility exercise only assessed whether participants engaged in these activities, frequency, intensity, and duration could not be evaluated; therefore, dose–response analyses for these two exercise components were not performed.

### 2.6 Covariates

Based on previous related studies and data availability, age group, sex, educational level, living arrangement, and marital status were included as covariates. To avoid sparse categories in the regression models, marital status was dichotomized as married and not married. Age was categorized into 65–69 years, 70–74 years, 75–79 years, and ≥80 years; sex was categorized as male or female; educational level was stratified according to years or level of formal education; and living arrangement was used to reflect the household living context of older adults (20). These variables were entered as covariates in the multivariable models to minimize, as far as possible, the influence of confounding on the estimated relationship between physical activity and frailty.

### 2.7 Disease burden

Disease burden was assessed on the basis of 14 chronic disease items in the questionnaire, including hypertension, diabetes, coronary heart disease or angina, history of myocardial infarction, stroke or cerebrovascular accident, chronic lung disease, asthma, arthritis-related diseases, chronic kidney disease, malignant tumor, hyperlipidemia, osteoporosis, emotional problems such as depression or anxiety, and other chronic diseases (16). These items were scored according to presence or absence and then summed to generate a disease burden score (disease score recalc), with a higher score indicating a greater cumulative burden of chronic disease. Given that the FRAIL scale already includes an item related to disease burden, this variable was not included in the primary analysis in order to avoid overadjustment for conceptually overlapping information. Instead, it was incorporated in the sensitivity analysis to test the robustness of the association between physical activity and frailty.

### 2.8 Exploratory assessment of multimorbidity heterogeneity

To better characterize heterogeneity within multimorbidity, the 14 chronic conditions were further classified into five disease-system categories: cardiovascular/metabolic conditions, cerebrovascular/neuropsychiatric conditions, respiratory conditions, musculoskeletal conditions, and renal/cancer/other conditions. A binary indicator was created for each disease-system category, and a disease-system count was calculated as the number of affected systems for each participant. These variables were used in exploratory supplementary analyses to describe disease-system distributions across frailty status groups and to examine whether the association between physical activity level and frailty status remained robust after additional adjustment for disease burden, disease-system count, and disease-system indicators.

### 2.9 Bias control and data processing

To improve data quality and reduce information bias, the raw data underwent systematic cleaning and consistency checks before formal analysis. First, the coding direction of all variables was standardized to ensure consistent meanings across items during scoring and statistical analysis. Second, the “other chronic diseases” item was dichotomized, and the disease burden score was recalculated accordingly to improve the accuracy and comparability of disease-related information. Third, missing values and logical consistency were checked for key variables, with particular attention to possible contradictions or abnormal records across physical activity items, frailty items, and chronic disease items. Questionnaires with serious logical conflicts that could not be verified were excluded, whereas variables with coding anomalies that could be corrected were recoded according to unified rules.

### 2.10 Statistical analysis

Descriptive analyses were conducted for the basic characteristics of the sample. Categorical variables were presented as frequencies and percentages, and between-group comparisons were performed using the chi-square test or Fisher’s exact test, depending on data distribution and cell counts. Continuous or count variables were described using mean ± standard deviation or median (interquartile range), as appropriate according to their distribution, and between-group comparisons were conducted using the Kruskal–Wallis test. Based on frailty grouping, stratified comparisons were made for age group, sex, educational level, living arrangement, marital status, regular exercise, disease burden, and physical activity characteristics, and contingency tables were constructed to examine the relationships of physical activity level, strength training, and flexibility exercise with frailty status. For physical activity level, an ordinal categorical variable, a trend test was further performed to assess whether a dose–response relationship existed between activity level and frailty status.

In the multivariable analysis, frailty group was treated as the dependent variable, and ordinal logistic regression was used to examine the associations between physical activity characteristics and frailty status. The models were built hierarchically. Model 1 included physical activity level only. Model 2 additionally included strength training and flexibility exercise. Model 3 further adjusted for age group, sex, educational level, and living arrangement. Model 4 additionally adjusted for marital status and was considered the extended multivariable-adjusted model. Results are presented as odds ratios (ORs) with 95% confidence intervals (CIs). To assess the robustness of the findings, sensitivity analyses were conducted. Spearman correlation analysis was used to examine the correlations among physical activity score, disease burden score, and total frailty score. In addition, linear regression models were fitted using total frailty score as a continuous dependent variable, and disease burden score was further added in an extended model to evaluate whether the association between physical activity and frailty remained after controlling for the cumulative burden of chronic disease. All tests were two-sided, and *p* < 0.05 was considered statistically significant.

Because of the cross-sectional design, all regression estimates were interpreted as associations with current frailty status rather than as evidence of incident frailty or causal effects. Additional exploratory analyses were conducted to address multimorbidity heterogeneity. Sensitivity analyses further adjusted for total disease burden, disease-system count, and disease-system indicators to evaluate the robustness of the association between physical activity level and frailty status. The proportional odds assumption for the ordinal logistic regression models was assessed.

Available sociodemographic, behavioral, and clinical determinants, including age group, sex, education, living arrangement, marital status, disease burden, and disease-system categories, were considered in the main and sensitivity analyses. Because the frail group was relatively small, formal interaction analyses or multiple subgroup analyses were not performed.

### 2.11 Ethics

The study was conducted in accordance with the principles of the Declaration of Helsinki (21). Before the survey began, all participants were fully informed of the study purpose, survey content, and intended use of the data, and participated on the basis of informed and voluntary consent. The questionnaire survey was conducted anonymously, and all collected data were used solely for scientific research and analysis. Participant privacy and data security were strictly protected throughout the study.

## 3 Results

### 3.1 Sample characteristics

A total of 500 community-dwelling older adults with multimorbidity were included in this study (Table 1), comprising 200 non-frail individuals (40.0%), 238 prefrail individuals (47.6%), and 62 frail individuals (12.4%). In the overall sample, women accounted for the majority (59.8%), 38.6% were aged 75 years or older, 10.4% lived alone, and the median number of chronic conditions was 3.0 (IQR: 2.0–4.0). Stratified comparisons showed that greater frailty severity was associated with older age overall: the proportion of participants aged ≥80 years was markedly higher in the frail group, whereas those aged 65–69 years were primarily concentrated in the non-frail group (*p* < 0.001). Educational level and living arrangement also differed across frailty categories, with the frail group showing relatively higher proportions of individuals without formal education and those living with their children (both *p* < 0.01). Regarding marital status, married individuals were more common in the non-frail group, whereas widowed individuals accounted for a higher proportion of the frail group (*p* = 0.025). The frail group had the lowest proportion of regular exercisers and the highest chronic disease burden, with a median number of chronic conditions of 5.0 (IQR: 4.0–5.8), which was substantially higher than that of the non-frail group [2.0 (IQR: 2.0–3.0)] (*p* < 0.001). Differences in physical activity level were particularly pronounced across the three groups: individuals meeting the recommended activity level were more frequently observed in the non-frail group, whereas inactive or only occasionally active individuals were mainly concentrated in the frail group (*p* < 0.001). In contrast, neither strength training nor flexibility exercise differed significantly across frailty categories. However, the frail group included only 62 participants, and some exposure categories contained few frail participants; therefore, minimum detectable effect sizes for the main comparisons were further reported in Supplementary Table S1.

**Table 1:** Participant characteristics overall and by frailty status.

Variable	Category	Overall	Non-frail	Pre-frail	Frail	Statistic	*p* value
Age group	65–69 years	156 (31.2)	96 (48.0)	58 (24.4)	2 (3.2)	χ^2^ = 135.733	<0.001
	70–74 years	151 (30.2)	71 (35.5)	73 (30.7)	7 (11.3)		
	75–79 years	107 (21.4)	27 (13.5)	62 (26.1)	18 (29.0)		
	≥80 years	86 (17.2)	6 (3.0)	45 (18.9)	35 (56.5)		
Sex	Male	201 (40.2)	77 (38.5)	93 (39.1)	31 (50.0)	χ^2^ = 2.843	0.241
	Female	299 (59.8)	123 (61.5)	145 (60.9)	31 (50.0)		
Educational level	No formal education	61 (12.2)	15 (7.5)	33 (13.9)	13 (21.0)	χ^2^ = 20.916	0.007
	Primary school	137 (27.4)	55 (27.5)	59 (24.8)	23 (37.1)		
	Junior high school	138 (27.6)	54 (27.0)	73 (30.7)	11 (17.7)		
	High school/technical secondary school	97 (19.4)	39 (19.5)	48 (20.2)	10 (16.1)		
	College degree or above	67 (13.4)	37 (18.5)	25 (10.5)	5 (8.1)		
Living arrangement	Living alone	52 (10.4)	25 (12.5)	23 (9.7)	4 (6.5)	χ^2^ = 24.158	0.002
	Living with spouse	155 (31.0)	77 (38.5)	60 (25.2)	18 (29.0)		
	Living with children	132 (26.4)	36 (18.0)	70 (29.4)	26 (41.9)		
	Living with spouse and children	119 (23.8)	47 (23.5)	60 (25.2)	12 (19.4)		
	Other	42 (8.4)	15 (7.5)	25 (10.5)	2 (3.2)		
Marital status	Never married	6 (1.2)	2 (1.0)	3 (1.3)	1 (1.6)	G^2^ = 14.460	0.025
	Married	314 (62.8)	140 (70.0)	144 (60.5)	30 (48.4)		
	Divorced/Separated	23 (4.6)	9 (4.5)	13 (5.5)	1 (1.6)		
	Widowed	157 (31.4)	49 (24.5)	78 (32.8)	30 (48.4)		
Regular exercise	Yes	199 (39.8)	96 (48.0)	91 (38.2)	12 (19.4)	χ^2^ = 16.673	<0.001
	No	301 (60.2)	104 (52.0)	147 (61.8)	50 (80.6)		
Number of chronic conditions	Median (IQR)	3.0 (2.0–4.0)	2.0 (2.0–3.0)	3.0 (2.0–4.0)	5.0 (4.0–5.8)	H = 131.178	<0.001
Physical activity level	Inactive/occasionally active	97 (19.4)	13 (6.5)	45 (18.9)	39 (62.9)	χ^2^ = 119.707	<0.001
	Regularly active but below the recommended level	276 (55.2)	106 (53.0)	150 (63.0)	20 (32.3)		
	Meeting the recommended level	127 (25.4)	81 (40.5)	43 (18.1)	3 (4.8)		
Strength training	No	369 (73.8)	143 (71.5)	174 (73.1)	52 (83.9)	χ^2^ = 3.858	0.145
	Yes	131 (26.2)	57 (28.5)	64 (26.9)	10 (16.1)		
Flexibility exercise	No	332 (66.4)	127 (63.5)	166 (69.7)	39 (62.9)	χ^2^ = 2.289	0.318
	Yes	168 (33.6)	73 (36.5)	72 (30.3)	23 (37.1)		

### 3.2 Exploratory analysis of multimorbidity heterogeneity

Exploratory analyses showed clear heterogeneity in disease-system burden across frailty status groups. Cardiovascular/metabolic conditions were highly prevalent in all groups, whereas cerebrovascular/neuropsychiatric, musculoskeletal, and renal/cancer/other conditions were more common among frail participants. Specifically, the prevalence of cerebrovascular/neuropsychiatric conditions increased from 13.0% in the non-frail group to 25.2% in the prefrail group and 38.7% in the frail group; musculoskeletal conditions increased from 33.5 to 50.0 and 77.4%, respectively; and renal/cancer/other conditions increased from 17.0 to 25.6 and 45.2%, respectively. The disease-system count also increased across frailty categories, supporting the presence of multimorbidity heterogeneity within the study population (Supplementary Table S3; Supplementary Figure S1).

### 3.3 Distribution of physical activity characteristics across frailty status

**Figure 1:**
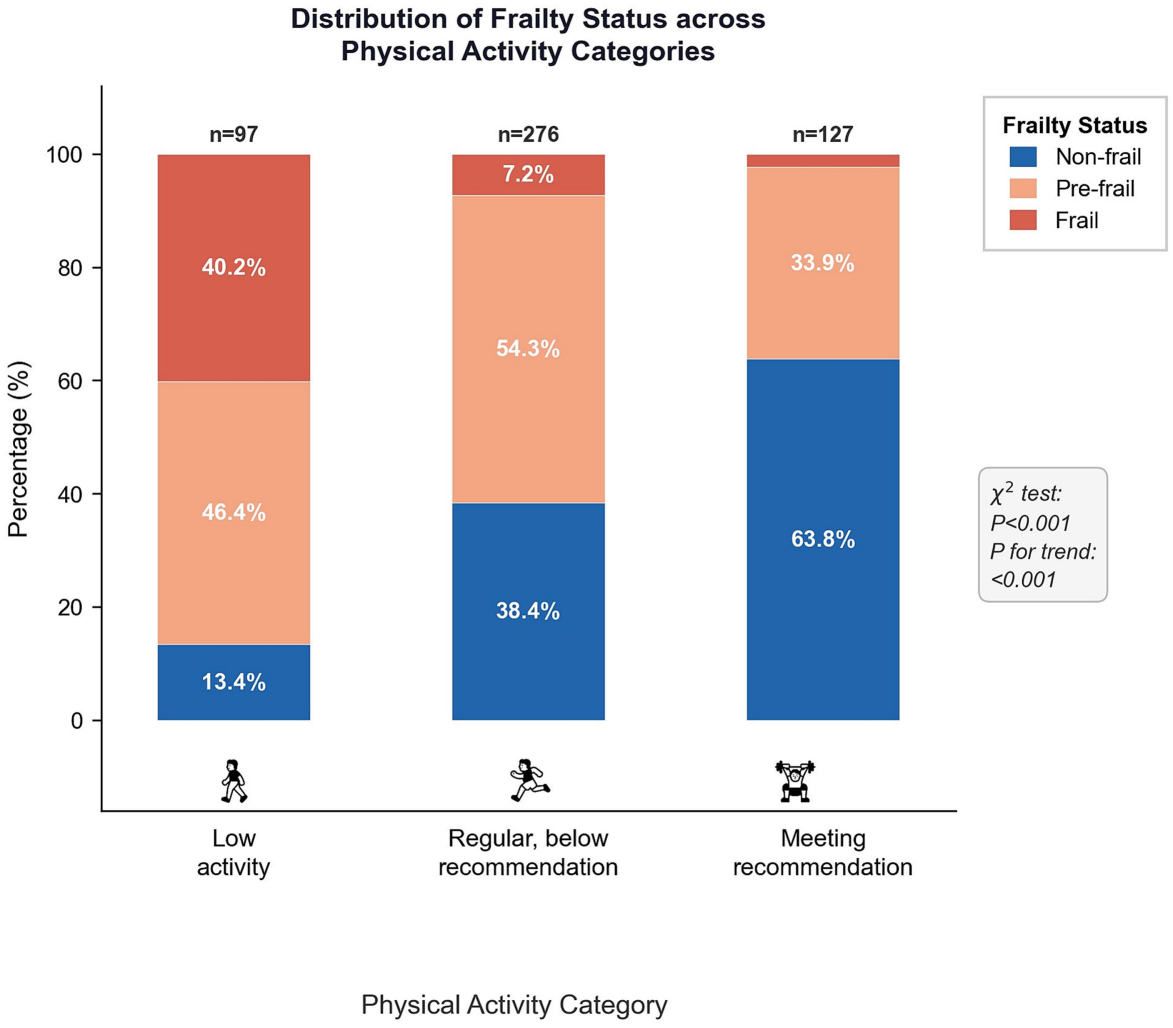
Frailty status across physical activity categories.

The distribution of physical activity characteristics varied markedly across frailty categories. Overall, 19.4% of participants were classified as low active, 55.2% as regularly active but not meeting the recommended level, and 25.4% as meeting the recommended level. Stratified comparisons showed that the frail group was predominantly concentrated in the low-activity category: 62.9% of frail individuals were classified as low active, whereas only 4.8% met the recommended activity level. In contrast, 40.5% of the non-frail group met the recommended level, and only 6.5% were low active. The prefrail group was primarily distributed in the regularly active but insufficient category (63.0%), representing an intermediate pattern between low and higher activity levels. The chi-square test indicated a significant association between physical activity level and frailty status, with a clear linear trend, such that frailty severity progressively decreased as physical activity level increased (all *p* < 0.001) (Figure 1). By comparison, based on binary participation indicators, the distributions of strength training and flexibility exercise across frailty categories did not reach statistical significance. Although the proportion of participants not engaging in strength training was relatively higher in the frail group (83.9%) than in the non-frail group (71.5%), the overall difference remained limited. The distribution of flexibility exercise was also similar across the three groups. Taken together, the association between overall physical activity level and frailty status was more pronounced, whereas individual components such as strength training or flexibility exercise did not show clear stratified differences (see Table 2).

**Table 2:** Physical activity characteristics across frailty status groups.

Variable	Category	Overall	Non-frail	Pre-frail	Frail	Statistic	*p* value	Method	Trend test
Physical activity level	Low activity	97 (19.4%)	13 (6.5%)	45 (18.9%)	39 (62.9%)	χ^2^ = 119.707, df = 4	<0.001	Chi^2^	Trend χ^2^ = 91.893, *p* <0.001
	Regular but below recommendation	276 (55.2%)	106 (53.0%)	150 (63.0%)	20 (32.3%)				
	Meeting recommendation	127 (25.4%)	81 (40.5%)	43 (18.1%)	3 (4.8%)				
Strength exercise	No	369 (73.8%)	143 (71.5%)	174 (73.1%)	52 (83.9%)	χ^2^ = 3.858, df = 2	0.145	Chi^2^	
	Yes	131 (26.2%)	57 (28.5%)	64 (26.9%)	10 (16.1%)				
Flexibility exercise	No	332 (66.4%)	127 (63.5%)	166 (69.7%)	39 (62.9%)	χ^2^ = 2.289, df = 2	0.318	Chi^2^	
	Yes	168 (33.6%)	73 (36.5%)	72 (30.3%)	23 (37.1%)				

### 3.4 Multivariable associations between physical activity characteristics and frailty status

**Figure 2:**
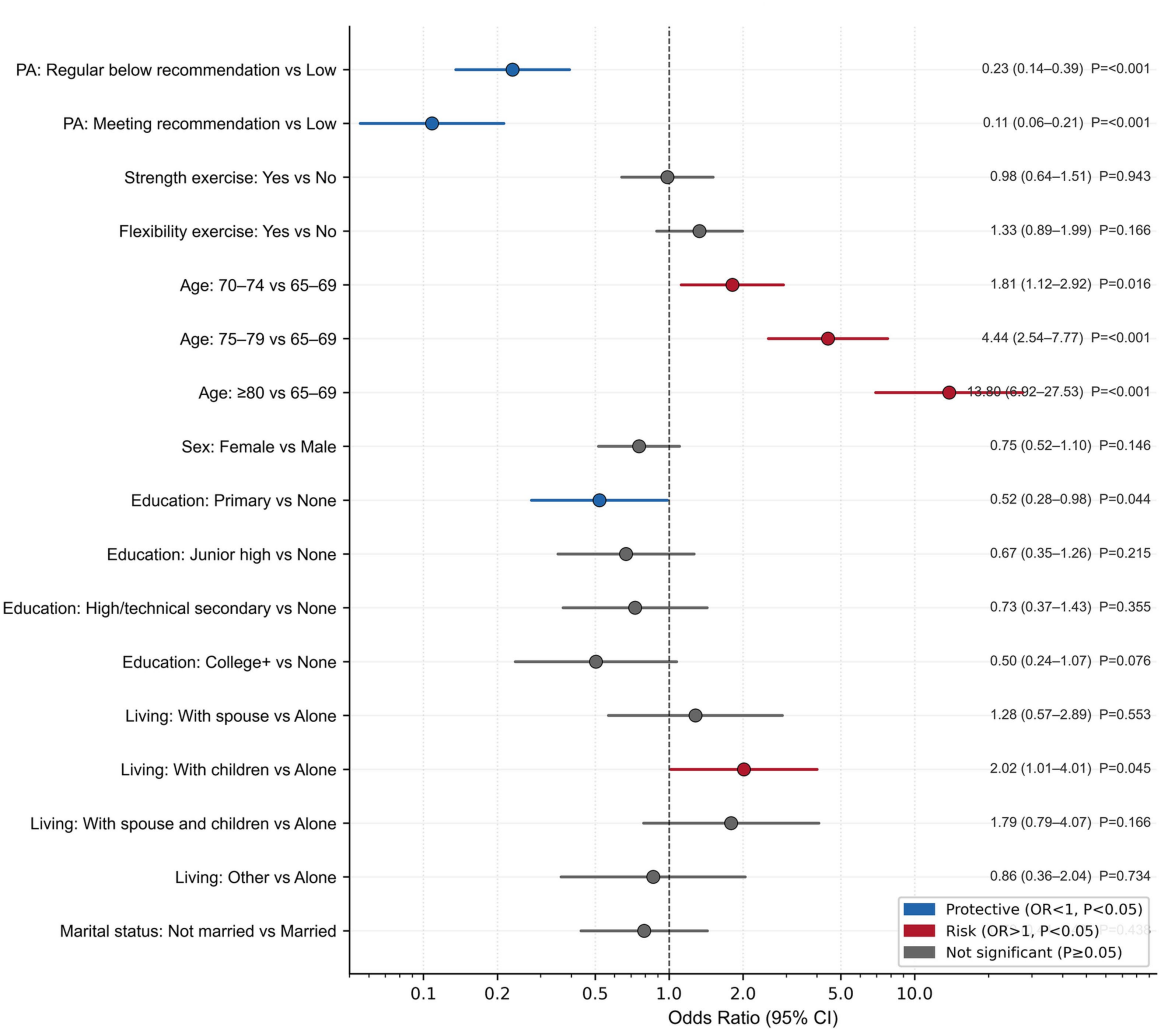
Associations of physical activity characteristics with frailty status.

In the ordinal logistic regression analysis (Table 3), physical activity level consistently showed a stable and clear inverse association with frailty status. Using low activity as the reference category, individuals who were regularly active but did not meet the recommended level had a substantially lower likelihood of being classified into a higher frailty category, regardless of whether strength training, flexibility exercise, and sociodemographic variables were included in the model. The association was even stronger among those who met the recommended activity level. Compared with the model including only physical activity level, the strength of this association was somewhat attenuated after additional adjustment for other physical activity characteristics, age, sex, educational level, and living arrangement; however, it remained clearly statistically significant. This finding suggests that the relationship between overall physical activity level and frailty is robust and cannot be fully explained by other factors. At the same time, physical activity level displayed an evident dose–response pattern: as activity level increased from low activity to regular activity and then to meeting the recommended level, frailty status progressively decreased. This indicates that even without reaching the recommended threshold, maintaining regular activity may already be associated with lower odds of higher frailty status. By contrast, the independent associations of strength training and flexibility exercise with frailty status did not reach statistical significance in the regression models, suggesting that, under the current binary measurement approach, no clear independent association was observed for these two specific exercise components (Figure 2).

**Table 3:** Associations of physical activity characteristics with frailty status in ordinal logistic regression models.

Variable	Model 1 OR (95% CI)	Model 1 P	Model 2 OR (95% CI)	Model 2 P	Model 3 OR (95% CI)	Model 3 P	Model 4 OR (95% CI)	Model 4 P
PA: Regular below recommendation vs. Low	0.15 (0.09–0.26)	<0.001	0.15 (0.09–0.24)	<0.001	0.23 (0.14–0.39)	<0.001	0.23 (0.14–0.39)	<0.001
PA: Meeting recommendation vs. Low	0.06 (0.03–0.10)	<0.001	0.05 (0.03–0.09)	<0.001	0.11 (0.06–0.22)	<0.001	0.11 (0.06–0.21)	<0.001
Strength exercise: Yes vs. No			1.04 (0.70–1.55)	0.854	0.99 (0.65–1.52)	0.968	0.98 (0.64–1.51)	0.943
Flexibility exercise: Yes vs. No			1.34 (0.92–1.95)	0.133	1.32 (0.88–1.97)	0.178	1.33 (0.89–1.99)	0.166
Age: 70–74 vs. 65–69					1.80 (1.11–2.90)	0.017	1.81 (1.12–2.92)	0.016
Age: 75–79 vs. 65–69					4.37 (2.50–7.64)	<0.001	4.44 (2.54–7.77)	<0.001
Age: ≥ 80 vs. 65–69					13.06 (6.65–25.65)	<0.001	13.80 (6.92–27.53)	<0.001
Sex: Female vs. Male					0.75 (0.51–1.09)	0.13	0.75 (0.52–1.10)	0.146
Education: Primary vs. None					0.51 (0.27–0.97)	0.039	0.52 (0.28–0.98)	0.044
Education: Junior high vs. None					0.65 (0.35–1.23)	0.187	0.67 (0.35–1.26)	0.215
Education: High/technical secondary vs. None					0.71 (0.36–1.40)	0.33	0.73 (0.37–1.43)	0.355
Education: College+ vs. None					0.50 (0.23–1.05)	0.068	0.50 (0.24–1.07)	0.076
Living: With spouse vs. Alone					1.54 (0.78–3.01)	0.212	1.28 (0.57–2.89)	0.553
Living: With children vs. Alone					2.07 (1.04–4.10)	0.037	2.02 (1.01–4.01)	0.045
Living: With spouse and children vs. Alone					2.14 (1.07–4.27)	0.031	1.79 (0.79–4.07)	0.166
Living: Other vs. Alone					0.90 (0.38–2.12)	0.804	0.86 (0.36–2.04)	0.734
Marital status: Not married vs. Married							0.79 (0.44–1.43)	0.438
P for trend for physical activity level		<0.001		<0.001		<0.001		<0.001

Analysis of covariates further showed that age remained an important correlate of frailty and exhibited a clear gradient effect: the likelihood of being classified into a higher frailty category increased progressively with advancing age, with particularly marked risk elevations among those aged 75 years and older. No significant association was observed between sex and frailty status. With respect to educational level, compared with individuals without formal education, those with higher educational attainment generally showed a lower tendency toward frailty; this difference reached statistical significance in the primary school education group, whereas the remaining education groups showed the same direction of association but did not all achieve statistical significance. Regarding living arrangement, living with children was associated with higher odds of being classified into a higher frailty category compared with living alone in Model 4 (OR = 2.02, 95% CI: 1.01–4.01, *p* = 0.045), whereas living with spouse, living with spouse and children, and other living arrangements were not statistically significant. Because living arrangement and frailty were measured at the same time point, this finding should be interpreted as a cross-sectional association. This finding suggests that the relationship between family co-residence patterns and frailty may be more complex, potentially reflecting both increased dependence on family support following functional decline and the influence of household caregiving arrangements. These multivariable results were consistent with the preceding distributional analyses, indicating that among community-dwelling older adults with multimorbidity, higher physical activity levels remained associated with lower frailty status after adjustment for available covariates.

**Figure 3:**
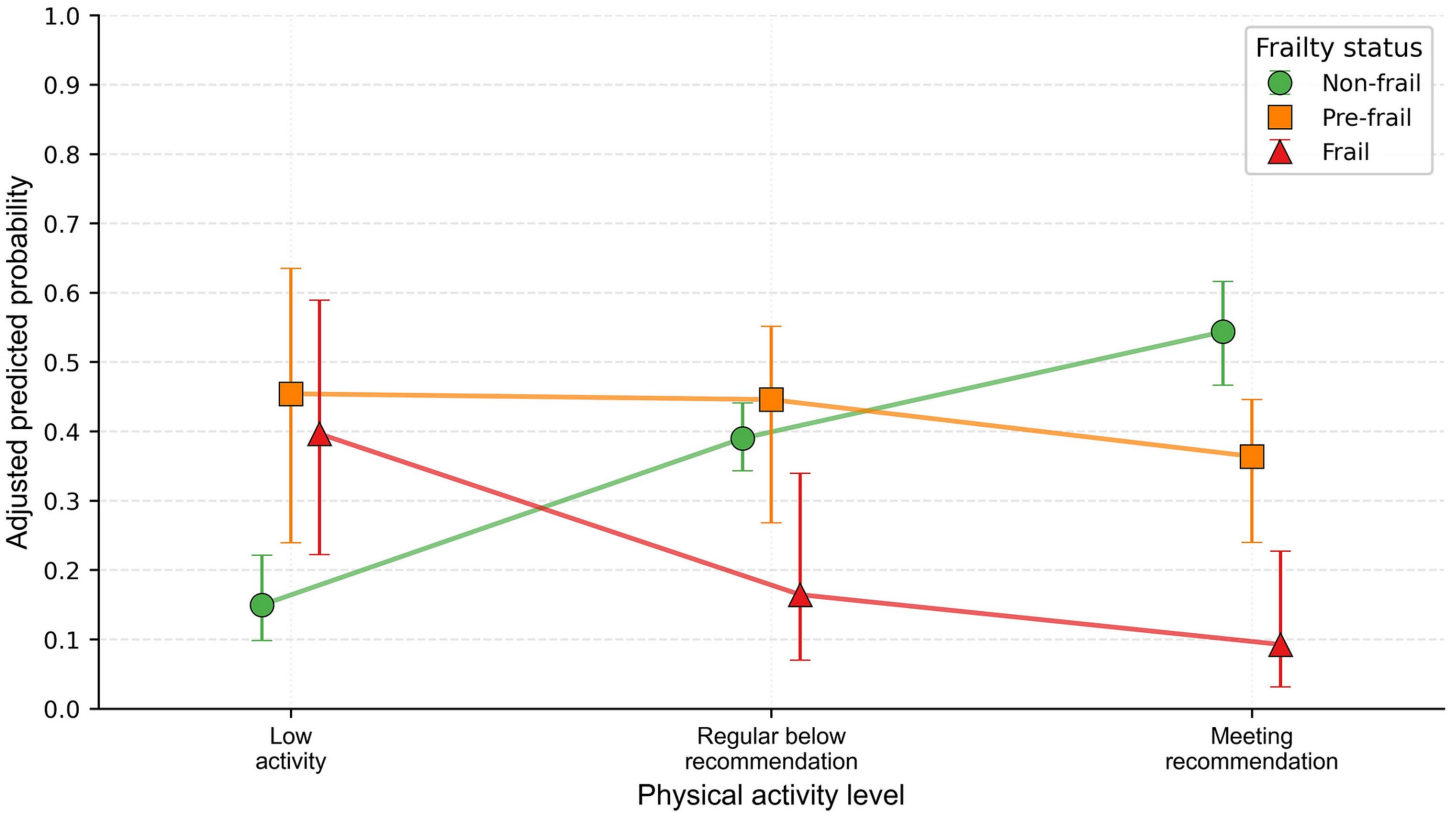
Adjusted predicted probabilities of frailty status across physical activity categories.

After additional adjustment for marital status in Model 4, the inverse association between physical activity level and frailty status remained statistically significant. Compared with participants with low activity, those who were regularly active but below the recommended level had lower odds of being classified into a higher frailty category (OR = 0.23, 95% CI: 0.14–0.39), and those meeting the recommended level showed an even stronger association (OR = 0.11, 95% CI: 0.06–0.21; both *p* < 0.001). The adjusted predicted probability plot further illustrated this graded pattern: the probability of frailty was highest among participants with low activity and decreased progressively across regular activity below the recommended level and meeting the recommended level, whereas the probability of being non-frail increased across these categories (Figure 3). Strength training and flexibility exercise still showed no independent statistical association after adjustment for available covariates. Marital status was not significantly associated with frailty status in the extended model. The proportional odds assumption was not violated in any ordinal logistic regression model, including the extended Model 4 (χ^2^ = 19.37, df = 17, *p* = 0.308; Supplementary Table S2).

### 3.5 Sensitivity analysis

The sensitivity analyses were consistent with the main findings. Spearman correlation analysis showed that physical activity score was moderately negatively correlated with total frailty score, whereas disease burden score was moderately positively correlated with total frailty score, the association between physical activity score and total frailty score remained significant after adjustment for marital status and other available covariates, and also persisted after further adjustment for disease burden. (Table 4). In contrast, the correlations of strength training and flexibility exercise with total frailty score were weak and did not reach statistical significance. In the linear regression analyses, physical activity score consistently maintained a stable inverse association with total frailty score and remained statistically significant in the unadjusted, partially adjusted, and extended multivariable-adjusted models, indicating that this relationship did not depend on age, sex, educational level, or living arrangement. Flexibility exercise reached significance only transiently in the partially adjusted model, but this association disappeared after further adjustment; therefore, no stable independent association was observed under the current binary measurement approach. Strength training showed no significant association in any of the models.

**Table 4:** Sensitivity analyses for the association between physical activity and frailty severity.

Panel	Variable	Model A1 Estimate (95% CI)	Model A1 P	Model A2 Estimate (95% CI)	Model A2 P	Model A3 Estimate (95% CI)	Model A3 P	Model B Estimate (95% CI)	Model B P	Note
A. Spearman correlation	PA score (1-7) vs. Frail total	rho = −0.454	<0.001							Spearman rho
A. Spearman correlation	Disease burden vs. Frail total	rho = 0.468	<0.001							Spearman rho
A. Spearman correlation	Strength exercise vs. Frail total	rho = −0.072	0.107							Spearman rho
A. Spearman correlation	Flexibility exercise vs. Frail total	rho = −0.053	0.24							Spearman rho
B. Linear regression	PA score continuous (1-7)	−0.316 (−0.366, −0.265)	<0.001	−0.329 (−0.382, −0.277)	<0.001	−0.225 (−0.280, −0.169)	<0.001	−0.078 (−0.132, −0.024)	0.005	OLS beta
B. Linear regression	Strength exercise: Yes vs. No			0.020 (−0.187, 0.228)	0.848	−0.006 (−0.200, 0.188)	0.951	0.059 (−0.111, 0.228)	0.495	OLS beta
B. Linear regression	Flexibility exercise: Yes vs. No			0.199 (0.003, 0.394)	0.046	0.148 (−0.035, 0.332)	0.114	0.045 (−0.115, 0.206)	0.58	OLS beta
B. Linear regression	Age: 70–74 vs. 65–69					0.152 (−0.066, 0.369)	0.172	0.110 (−0.079, 0.300)	0.253	OLS beta
B. Linear regression	Age: 75–79 vs. 65–69					0.594 (0.342, 0.847)	<0.001	0.581 (0.362, 0.801)	<0.001	OLS beta
B. Linear regression	Age: ≥ 80 vs. 65–69					1.179 (0.881, 1.476)	<0.001	1.003 (0.743, 1.264)	<0.001	OLS beta
B. Linear regression	Sex: Female vs. Male					−0.160 (−0.333, 0.012)	0.068	−0.078 (−0.229, 0.073)	0.31	OLS beta
B. Linear regression	Education: Primary vs. None					−0.303 (−0.595, −0.012)	0.041	−0.260 (−0.514, −0.006)	0.045	OLS beta
B. Linear regression	Education: Junior high vs. None					−0.229 (−0.523, 0.066)	0.128	−0.274 (−0.531, −0.018)	0.036	OLS beta
B. Linear regression	Education: High/technical secondary vs. None					−0.129 (−0.439, 0.182)	0.416	−0.320 (−0.592, −0.048)	0.021	OLS beta
B. Linear regression	Education: College+ vs. None					−0.152 (−0.492, 0.188)	0.38	−0.290 (−0.586, 0.007)	0.056	OLS beta
B. Linear regression	Living: With spouse vs. Alone					0.160 (−0.207, 0.526)	0.392	0.184 (−0.135, 0.503)	0.258	OLS beta
B. Linear regression	Living: With children vs. Alone					0.336 (0.029, 0.644)	0.032	0.353 (0.086, 0.621)	0.01	OLS beta
B. Linear regression	Living: With spouse and children vs. Alone					0.320 (−0.050, 0.691)	0.09	0.311 (−0.011, 0.634)	0.058	OLS beta
B. Linear regression	Living: Other vs. Alone					−0.196 (−0.591, 0.198)	0.328	−0.054 (−0.398, 0.291)	0.76	OLS beta
B. Linear regression	Marital status: Not married vs. Married					−0.052 (−0.318, 0.214)	0.702	−0.037 (−0.269, 0.194)	0.751	OLS beta
C. Additional disease adjustment	Disease burden score							0.476 (0.401, 0.550)	<0.001	OLS beta

After the inclusion of disease burden, the inverse association between physical activity score and total frailty score was attenuated but remained statistically significant, indicating that the relationship between higher physical activity levels and lower frailty severity retained a certain degree of independence. Meanwhile, disease burden score itself was significantly positively associated with total frailty score, suggesting that the accumulation of chronic conditions is an important correlate of frailty. The age effect also remained stable, with older adults aged 75 years and above having significantly higher total frailty scores than those aged 65–69 years. Educational attainment generally showed a protective pattern, and some education groups demonstrated lower frailty levels after further adjustment. Regarding living arrangement, participants living with their children or with both spouse and children had relatively higher total frailty scores, which was directionally consistent with the main model. Overall, the sensitivity analyses repeatedly confirmed the inverse relationship between physical activity and frailty and showed that this association persisted even after controlling for disease burden.

Additional ordinal logistic regression sensitivity analyses further supported these findings. After adjustment for total disease burden, participants who were regularly active but below the recommended level remained less likely to be classified into a higher frailty category than those with low activity levels (OR = 0.47, 95% CI: 0.27–0.83), as did those meeting the recommended activity level (OR = 0.34, 95% CI: 0.16–0.70). Similar associations were observed after additional adjustment for disease-system count and for specific disease-system indicators. Disease burden and disease-system count were themselves strongly associated with higher frailty status, indicating that multimorbidity burden is an important correlate of frailty while not fully explaining the association between physical activity level and frailty status (Supplementary Table S4).

## 4 Discussion

In this cross-sectional study of 500 community-dwelling older adults with multimorbidity, we found a significant and stable inverse association between physical activity level and frailty status. This association showed a graded pattern across descriptive analyses, ordinal logistic regression models, and sensitivity analyses. However, because physical activity, chronic conditions, and frailty were measured at the same time point, these findings should be interpreted as cross-sectional associations rather than evidence that physical activity prevents frailty onset or progression.

Frailty is shaped by multiple intersecting determinants, including sociodemographic characteristics, chronic disease burden, living context, health behaviors, and community environment. In the present study, we adjusted for available factors, including age, sex, education, living arrangement, marital status, and multimorbidity-related indicators. However, other relevant determinants, such as smoking, alcohol use, socioeconomic status, cognitive function, urban/rural residence, green space accessibility, and pollution exposure, were not available. Therefore, the findings should be interpreted as associations adjusted for available covariates, rather than as conclusions accounting for all intersectional determinants.

The type, number, severity, and combinations of chronic diseases may change over time, and these temporal changes cannot be captured in the present cross-sectional design. Longitudinal studies are better suited to distinguish baseline multimorbidity patterns from subsequent frailty onset. Tazzeo et al. (13) showed that multimorbidity patterns were differentially associated with incident physical frailty, with cardiovascular and neuropsychiatric patterns being particularly relevant. In contrast, the present study used multimorbidity as a high-risk population context and focused on the association between current physical activity characteristics and current frailty status. To partially address heterogeneity within multimorbidity, we added exploratory analyses based on disease-system categories and disease-system count. These analyses showed that frail participants had a higher burden of several disease systems, particularly cerebrovascular/neuropsychiatric, musculoskeletal, and renal/cancer/other conditions, and that the association between physical activity level and frailty status remained robust after further adjustment for disease burden and disease-system indicators. Nevertheless, these supplementary analyses cannot replace longitudinal evidence and should be interpreted as cross-sectional characterization.

The core feature of frailty lies in reduced physiological reserve and diminished resilience to stress, and its onset and progression are closely related to chronic inflammation, loss of muscle mass and strength, impaired mitochondrial function, and abnormalities in metabolic regulation (22). Previous reviews have indicated that regular exercise may intervene in frailty-related pathways through multiple mechanisms, including reducing inflammatory responses, improving insulin sensitivity, enhancing anabolic processes and muscle protein synthesis, and promoting autophagy and mitochondrial function (23, 33). Aerobic exercise and resistance-based activities correspond to different domains, such as endurance, walking capacity, muscle strength, and functional performance, while multicomponent exercise programs often address several core dimensions of frailty simultaneously (11). Among older adults with multimorbidity, randomized controlled trials have shown that low-impact, moderate-intensity exercise can improve fatigue, physical activity level, and frailty-related outcomes, suggesting that even in populations with substantial disease burden and limited functional reserve, regular activity at an appropriate intensity can still provide tangible benefits (24).

The association between living arrangement and frailty should be interpreted cautiously. Living with children may not be a causal determinant of frailty; rather, older adults who are already frail, functionally dependent, or in need of care may be more likely to live with their children. Because the timing and reasons for changes in living arrangement were not collected, reverse causation cannot be excluded. Therefore, living arrangement should be regarded as a contextual correlate of frailty rather than a causal factor.

Strength training and flexibility exercise did not show independent statistical associations after adjustment in the present study. This finding should not be interpreted as evidence that these activities are ineffective. Rather, it may reflect the limitations of measurement and study design. In the RAPA questionnaire, strength training and flexibility exercise are each assessed by a single binary item, indicating only whether participants engaged in the activity, without information on frequency, intensity, duration, progression, or adherence. Therefore, true dose–response associations could not be examined. Similar inconsistencies have been reported in previous work: some isolated physical performance components, such as grip strength or flexibility tests, were not consistently associated with frailty trajectories or did not show significant improvement, whereas multicomponent exercise programs showed clearer benefits. Intervention studies often prescribe structured exercise with defined intensity, frequency, duration, and progression, and usually combine aerobic, resistance, balance, and flexibility components (19, 25, 26). By contrast, our study captured self-reported participation in strength or flexibility exercise at a single time point. Thus, the non-significant findings for strength training and flexibility exercise are more likely related to crude exposure measurement, limited statistical power, and the observational cross-sectional design than to a lack of potential benefit (27).

The positive association between age and frailty status observed in this study is consistent with previous research, indicating that as age increases, physiological reserve, activity capacity, and resilience to stress decline progressively, thereby increasing the likelihood of transition from non-frailty to prefrailty and eventually frailty (28, 29). Older adults with higher educational attainment exhibited lower frailty severity. Previous cohort studies have shown that lower educational level is associated with more adverse frailty trajectories, whereas community-dwelling older adults with higher health literacy are more likely to maintain a non-frail status, suggesting that education may exert a protective effect through better access to health information, stronger disease management capacity, and the long-term accumulation of healthier behaviors (30, 31). In this study, frailty distribution differed according to living arrangement, but this finding should be interpreted with caution. The relationship between living arrangement and frailty is complex and is often influenced simultaneously by sex, social participation, family support, and cultural background, and findings across studies have not been entirely consistent (28, 30). The phenomenon observed here therefore reflects an association rather than a simple causal relationship. One possible explanation is that older adults with poorer functional status or greater care needs are more likely to live with their children as part of family caregiving arrangements. Previous studies have also suggested that as parental frailty increases, adult children become correspondingly more likely to participate in caregiving (32).

From the perspective of Chinese community health practice, these findings may be translated into a staged physical activity promotion strategy within existing community service systems. For older adults with low activity levels, family doctor teams and community health service centers could identify high-risk individuals during chronic disease follow-up, annual health assessments, and health education activities, and provide brief advice to help them reduce inactivity and begin regular low-intensity activity. For those who are regularly active but still below the recommended level, community programs could gradually increase activity frequency, duration, or intensity through group walking, exercise classes, or activities organized in community senior centers. For those already meeting the recommended level, the priority should be maintaining activity and preventing decline during disease fluctuations, hospitalization, or caregiving changes. These staged recommendations are consistent with China’s family doctor contract services and older-adult health management system, which emphasize health management, health education, follow-up, and stratified services for older adults and chronic disease populations. However, because the present study was cross-sectional, these recommendations should be viewed as practical implications rather than evidence of intervention effectiveness. Future studies should also incorporate neighborhood-level environmental indicators, such as green space accessibility, walkability, and pollution exposure, to better understand how community environments shape physical activity and frailty among older adults with multimorbidity.

This study has several limitations. First, the cross-sectional design and absence of prospective follow-up preclude conclusions about temporal order or causality. Because physical activity, chronic conditions, living arrangement, and frailty were measured at the same time point, we could not determine whether lower physical activity preceded frailty, whether frailty reduced activity, or whether changes in multimorbidity or living status occurred before frailty onset. Second, physical activity and frailty were assessed mainly using self-reported questionnaires. In particular, strength training and flexibility exercise were measured only as binary participation indicators, without information on frequency, intensity, duration, progression, or adherence; therefore, dose–response analyses for these components could not be performed. Third, although we adjusted for available covariates and added exploratory analyses of disease burden and disease-system categories, several relevant factors were not collected, including smoking, alcohol use, socioeconomic status, cognitive function, urban/rural residence, green space accessibility, walkability, and pollution exposure. Residual confounding from behavioral, socioeconomic, cognitive, and environmental factors therefore cannot be excluded. Finally, the relatively small number of frail participants limited the statistical power for sparse exposure categories and prevented reliable subgroup or interaction analyses.

## 5 Conclusion

The findings of this study indicate that physical activity level was significantly associated with frailty status and demonstrated a significant dose–response relationship. Compared with low-active individuals, those who were regularly active but did not meet the recommended level, as well as those who achieved the recommended level, were both significantly less likely to be classified into a higher frailty category. Using the available binary indicators, strength training and flexibility exercise did not show independent statistical associations after adjustment for covariates. The sensitivity analyses were consistent with the main findings, showing that higher physical activity scores remained associated with lower total frailty scores, and that this association persisted even after further adjustment for disease burden. Within the limits of the available covariates and cross-sectional design, these results suggest that, among community-dwelling older adults with multimorbidity, overall physical activity level may serve as a useful behavioral marker for identifying individuals with higher current frailty burden and for guiding staged activity promotion within Chinese community health services. Therefore, we could not determine whether living with children preceded frailty or resulted from frailty-related care needs.

## Data Availability

The datasets analyzed during the current study are not publicly available due to privacy and confidentiality considerations involving the participants, but are available from the corresponding author on reasonable request.
