# Supplementary figures and images for: Association between physical activity level and frailty status among community-dwelling older adults with multimorbidity: a cross-sectional study
Source: Front Public Health. 2026 Jul 1;14:1826097. doi: 10.3389/fpubh.2026.1826097 (PMC13368540; doi:10.3389/fpubh.2026.1826097)

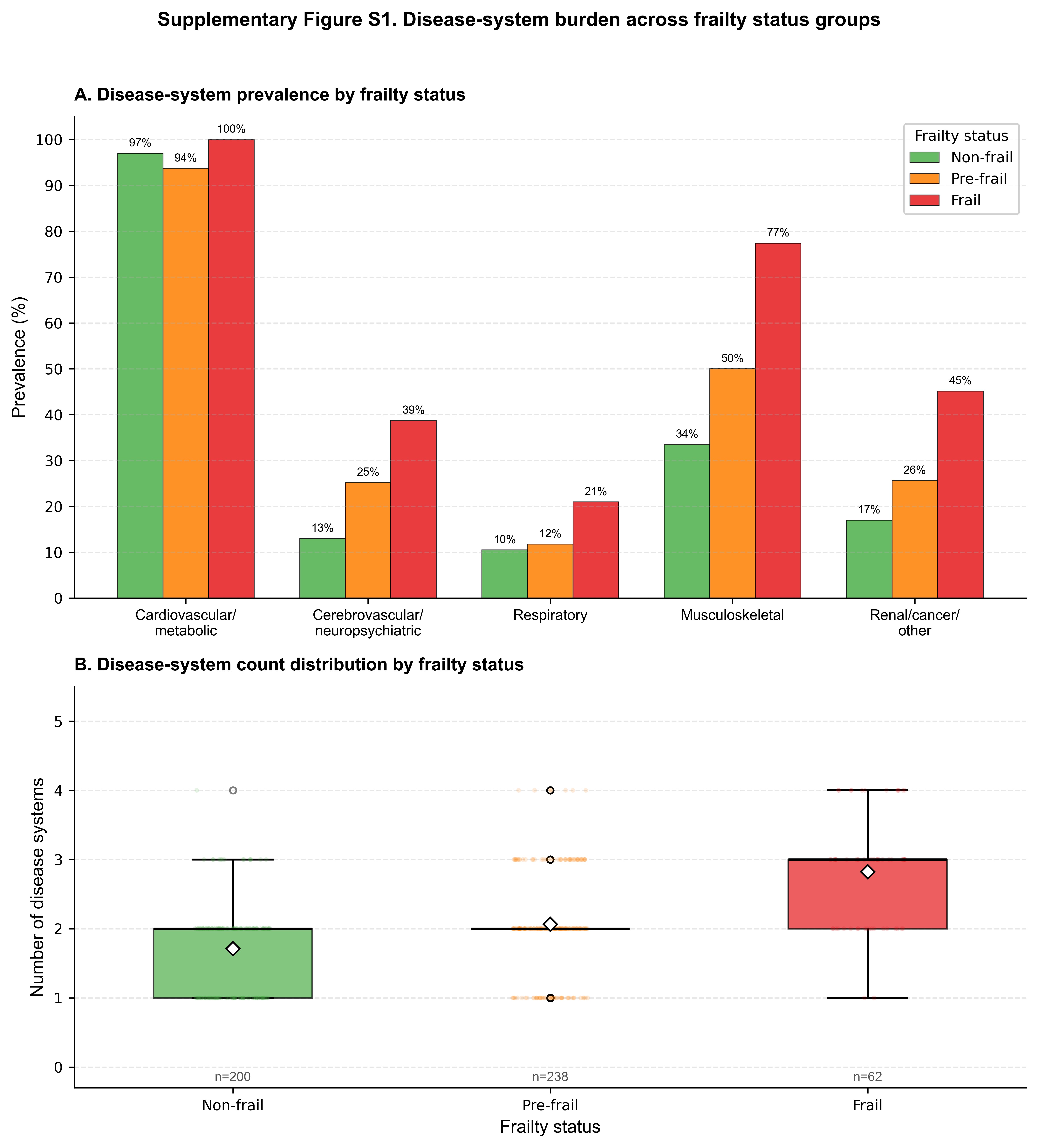

Supplement: Supplementary file 1 [file Figure_1.JPEG]

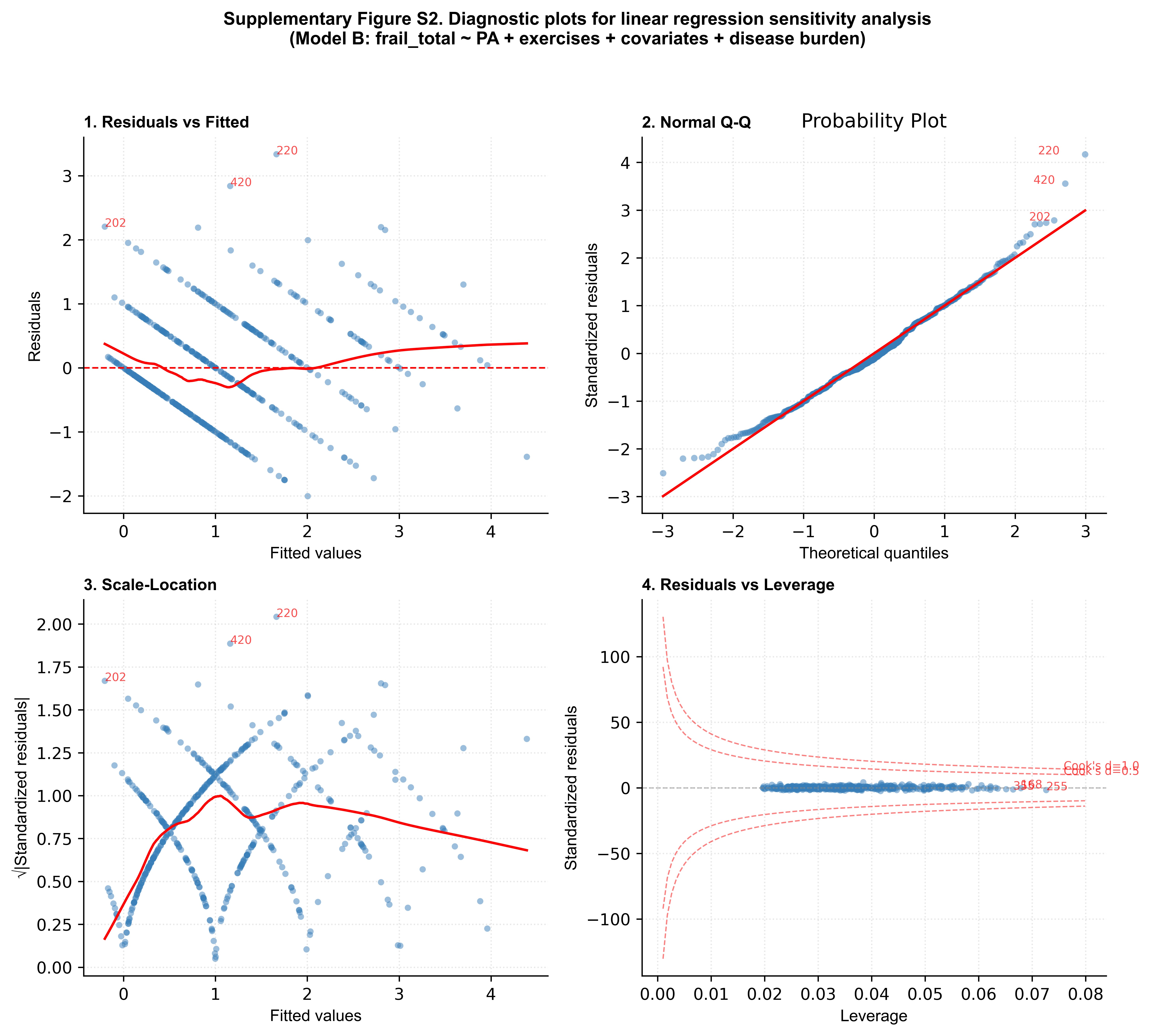

Supplement: Supplementary file 2 [file Figure_2.JPEG]
