# Supplementary material for: Association between physical activity level and frailty status among community-dwelling older adults with multimorbidity: a cross-sectional study
Source: Front Public Health. 2026 Jul 1;14:1826097. doi: 10.3389/fpubh.2026.1826097 (PMC13368540; doi:10.3389/fpubh.2026.1826097)
Supplement: Supplementary file 3 [file Table_1.DOCX]

Supplementary Table S1. Sample size, event distribution, and minimum detectable effect size

| **Section** | **Comparison** | **Reference group** | **Comparison group** | **n** | **Percentage** | **Frail events reference** | **Event rate reference** | **n comparison** | **Frail events comparison** | **Event rate comparison** | **Observed OR** | **Approximate minimum detectable OR** |
| --- | --- | --- | --- | --- | --- | --- | --- | --- | --- | --- | --- | --- |
| A. Sample size and event distribution | Total sample size | All |  | 500 | 100.0% |  |  |  |  |  |  |  |
|  | Frailty status | Non-frail |  | 200 | 40.0% |  |  |  |  |  |  |  |
|  |  | Pre-frail |  | 238 | 47.6% |  |  |  |  |  |  |  |
|  |  | Frail |  | 62 | 12.4% |  |  |  |  |  |  |  |
|  | Physical activity level | Low activity |  | 97 | 19.4% |  |  |  |  |  |  |  |
|  |  | Regularly active but below recommendation |  | 276 | 55.2% |  |  |  |  |  |  |  |
|  |  | Meeting recommendation |  | 127 | 25.4% |  |  |  |  |  |  |  |
|  | Strength exercise | No |  | 369 | 73.8% |  |  |  |  |  |  |  |
|  |  | Yes |  | 131 | 26.2% |  |  |  |  |  |  |  |
|  | Flexibility exercise | No |  | 332 | 66.4% |  |  |  |  |  |  |  |
|  |  | Yes |  | 168 | 33.6% |  |  |  |  |  |  |  |
|  | PA × Frailty: Low activity | Non-frail |  | 13 |  |  |  |  |  |  |  |  |
|  |  | Pre-frail |  | 45 |  |  |  |  |  |  |  |  |
|  |  | Frail |  | 39 |  |  |  |  |  |  |  |  |
|  | PA × Frailty: Regularly active but below recommendation | Non-frail |  | 106 |  |  |  |  |  |  |  |  |
|  |  | Pre-frail |  | 150 |  |  |  |  |  |  |  |  |
|  |  | Frail |  | 20 |  |  |  |  |  |  |  |  |
|  | PA × Frailty: Meeting recommendation | Non-frail |  | 81 |  |  |  |  |  |  |  |  |
|  |  | Pre-frail |  | 43 |  |  |  |  |  |  |  |  |
|  |  | Frail |  | 3 |  |  |  |  |  |  |  |  |
|  | Strength exercise No | Non-frail |  | 143 |  |  |  |  |  |  |  |  |
|  |  | Pre-frail |  | 174 |  |  |  |  |  |  |  |  |
|  |  | Frail |  | 52 |  |  |  |  |  |  |  |  |
|  | Strength exercise Yes | Non-frail |  | 57 |  |  |  |  |  |  |  |  |
|  |  | Pre-frail |  | 64 |  |  |  |  |  |  |  |  |
|  |  | Frail |  | 10 |  |  |  |  |  |  |  |  |
|  | Flexibility exercise No | Non-frail |  | 127 |  |  |  |  |  |  |  |  |
|  |  | Pre-frail |  | 166 |  |  |  |  |  |  |  |  |
|  |  | Frail |  | 39 |  |  |  |  |  |  |  |  |
|  | Flexibility exercise Yes | Non-frail |  | 73 |  |  |  |  |  |  |  |  |
|  |  | Pre-frail |  | 72 |  |  |  |  |  |  |  |  |
|  |  | Frail |  | 23 |  |  |  |  |  |  |  |  |
| B. Minimum detectable OR | Regular below recommendation vs Low activity | Low activity | Regular below recommendation |  |  | 39 | 0.4021 | 276 | 20 | 0.0725 | 0.12 | 1.94 |
|  | Meeting recommendation vs Low activity |  | Meeting recommendation |  |  | 39 | 0.4021 | 127 | 3 | 0.0236 | 0.04 | 2.14 |
|  | Strength exercise Yes vs No | No | Yes |  |  | 52 | 0.1409 | 131 | 10 | 0.0763 | 0.50 | 2.07 |
|  | Flexibility exercise Yes vs No |  | Yes |  |  | 39 | 0.1175 | 168 | 23 | 0.1369 | 1.19 | 2.06 |

Supplementary Table S2. Proportional odds assumption tests

| **Model** | **Test type** | **Variable** | **Chi-square statistic** | **df** | **P value** | **Interpretation** |
| --- | --- | --- | --- | --- | --- | --- |
| Model 1 | Global test | All variables | 3.24 | 2 | 0.198 | No evidence against proportional odds assumption |
| Model 2 | Global test | All variables | 8.34 | 4 | 0.08 | No evidence against proportional odds assumption |
| Model 3 | Global test | All variables | 17.85 | 16 | 0.3325 | No evidence against proportional odds assumption |
| Model 4 | Global test | All variables | 19.37 | 17 | 0.308 | No evidence against proportional odds assumption |

Supplementary Table S3

| **Section** | **Variable** | **Conditions included** | **Overall** | **Non-frail** | **Pre-frail** | **Frail** | **Statistic** | **P value** | **Method** | **Note** |
| --- | --- | --- | --- | --- | --- | --- | --- | --- | --- | --- |
| A. Disease-system indicators | Cardiovascular/metabolic | dis_htn, dis_dm, dis_chd, dis_mi, dis_hyperlipid | 479 (95.8%) | 194 (97.0%) | 223 (93.7%) | 62 (100.0%) | χ²=6.05 | 0.049 | Chi-square (some expected<5, interpret with caution) |  |
| A. Disease-system indicators | Cerebrovascular/neuropsychiatric | dis_stroke, dis_psych | 110 (22.0%) | 26 (13.0%) | 60 (25.2%) | 24 (38.7%) | χ²=20.96 | <0.001 | Chi-square test |  |
| A. Disease-system indicators | Respiratory | dis_lung, dis_asthma | 62 (12.4%) | 21 (10.5%) | 28 (11.8%) | 13 (21.0%) | χ²=4.94 | 0.084 | Chi-square test |  |
| A. Disease-system indicators | Musculoskeletal | dis_arthritis, dis_osteoporosis | 234 (46.8%) | 67 (33.5%) | 119 (50.0%) | 48 (77.4%) | χ²=38.54 | <0.001 | Chi-square test |  |
| A. Disease-system indicators | Renal/cancer/other | dis_ckd, dis_cancer, dis_other | 123 (24.6%) | 34 (17.0%) | 61 (25.6%) | 28 (45.2%) | χ²=20.50 | <0.001 | Chi-square test |  |
| A. Individual diseases | Hypertension | dis_htn | 346 (69.2%) | 132 (66.0%) | 162 (68.1%) | 52 (83.9%) | χ²=7.37 | 0.025 | Chi-square test |  |
| A. Individual diseases | Diabetes mellitus | dis_dm | 201 (40.2%) | 82 (41.0%) | 90 (37.8%) | 29 (46.8%) | χ²=1.73 | 0.421 | Chi-square test |  |
| A. Individual diseases | Coronary heart disease | dis_chd | 98 (19.6%) | 34 (17.0%) | 47 (19.7%) | 17 (27.4%) | χ²=3.27 | 0.195 | Chi-square test |  |
| A. Individual diseases | Myocardial infarction | dis_mi | 26 (5.2%) | 13 (6.5%) | 6 (2.5%) | 7 (11.3%) | χ²=8.82 | 0.012 | Chi-square (some expected<5, interpret with caution) |  |
| A. Individual diseases | Stroke | dis_stroke | 61 (12.2%) | 10 (5.0%) | 34 (14.3%) | 17 (27.4%) | χ²=24.05 | <0.001 | Chi-square test |  |
| A. Individual diseases | Chronic lung disease | dis_lung | 52 (10.4%) | 19 (9.5%) | 23 (9.7%) | 10 (16.1%) | χ²=2.50 | 0.287 | Chi-square test |  |
| A. Individual diseases | Asthma | dis_asthma | 11 (2.2%) | 3 (1.5%) | 5 (2.1%) | 3 (4.8%) | χ²=2.47 | 0.29 | Chi-square (some expected<5, interpret with caution) |  |
| A. Individual diseases | Arthritis | dis_arthritis | 150 (30.0%) | 33 (16.5%) | 80 (33.6%) | 37 (59.7%) | χ²=44.84 | <0.001 | Chi-square test |  |
| A. Individual diseases | Chronic kidney disease | dis_ckd | 38 (7.6%) | 5 (2.5%) | 19 (8.0%) | 14 (22.6%) | χ²=27.27 | <0.001 | Chi-square (some expected<5, interpret with caution) |  |
| A. Individual diseases | Cancer | dis_cancer | 37 (7.4%) | 8 (4.0%) | 19 (8.0%) | 10 (16.1%) | χ²=10.39 | 0.006 | Chi-square (some expected<5, interpret with caution) |  |
| A. Individual diseases | Hyperlipidemia | dis_hyperlipid | 205 (41.0%) | 81 (40.5%) | 82 (34.5%) | 42 (67.7%) | χ²=22.57 | <0.001 | Chi-square test |  |
| A. Individual diseases | Osteoporosis | dis_osteoporosis | 126 (25.2%) | 37 (18.5%) | 57 (23.9%) | 32 (51.6%) | χ²=27.91 | <0.001 | Chi-square test |  |
| A. Individual diseases | Psychiatric disorder | dis_psych | 55 (11.0%) | 16 (8.0%) | 28 (11.8%) | 11 (17.7%) | χ²=4.86 | 0.088 | Chi-square test |  |
| A. Individual diseases | Other chronic disease | dis_other | 63 (12.6%) | 21 (10.5%) | 29 (12.2%) | 13 (21.0%) | χ²=4.78 | 0.092 | Chi-square test |  |
| B. Disease burden summaries | Total disease count | 14 chronic conditions | 3 (2–4) | 2 (2–3) | 3 (2–4) | 5 (4–6) | H=131.18 | <0.001 | Kruskal-Wallis test | Median (IQR) |
| B. Disease burden summaries | Disease system count | 5 disease systems | 2 (2–2) | 2 (1–2) | 2 (2–2) | 3 (2–3) | H=93.07 | <0.001 | Kruskal-Wallis test | Median (IQR) |

Supplementary Table S4

| Variable | Base Model 4 OR (95% CI) | Base Model 4 P | Sensitivity Model 1 OR (95% CI) | Sensitivity Model 1 P | Sensitivity Model 2 OR (95% CI) | Sensitivity Model 2 P | Sensitivity Model 3 OR (95% CI) | Sensitivity Model 3 P | Note |
| --- | --- | --- | --- | --- | --- | --- | --- | --- | --- |
| PA: Regular below recommendation vs Low | 0.23 (0.14–0.39) | <0.001 | 0.47 (0.27–0.83) | 0.009 | 0.36 (0.21–0.62) | <0.001 | 0.35 (0.20–0.60) | <0.001 |  |
| PA: Meeting recommendation vs Low | 0.11 (0.06–0.21) | <0.001 | 0.34 (0.16–0.70) | 0.003 | 0.22 (0.11–0.44) | <0.001 | 0.22 (0.11–0.44) | <0.001 |  |
| Strength exercise: Yes vs No | 0.98 (0.64–1.51) | 0.943 | 1.14 (0.73–1.78) | 0.57 | 1.18 (0.75–1.84) | 0.479 | 1.18 (0.75–1.84) | 0.476 |  |
| Flexibility exercise: Yes vs No | 1.33 (0.89–1.99) | 0.166 | 1.24 (0.81–1.90) | 0.311 | 1.28 (0.85–1.94) | 0.243 | 1.22 (0.80–1.85) | 0.365 |  |
| Disease burden score |  |  | 2.81 (2.22–3.54) | <0.001 |  |  |  |  |  |
| Disease-system count |  |  |  |  | 2.81 (2.10–3.76) | <0.001 |  |  |  |
| Cardiovascular/metabolic conditions |  |  |  |  |  |  | 1.97 (0.76–5.12) | 0.164 |  |
| Cerebrovascular/neuropsychiatric conditions |  |  |  |  |  |  | 2.69 (1.67–4.33) | <0.001 |  |
| Respiratory conditions |  |  |  |  |  |  | 1.92 (1.04–3.54) | 0.037 |  |
| Musculoskeletal conditions |  |  |  |  |  |  | 3.67 (2.41–5.60) | <0.001 |  |
| Renal/cancer/other conditions |  |  |  |  |  |  | 2.60 (1.62–4.16) | <0.001 |  |
| P for trend for physical activity level |  | <0.001 |  | 0.005 |  | <0.001 |  | <0.001 | Trend test using pa_cat3 as continuous (1,2,3) |
